# Seasonal Abundance and Host-Feeding Patterns of Anopheline Vectors in Malaria Endemic Area of Iran

**DOI:** 10.1155/2010/671291

**Published:** 2010-09-13

**Authors:** Hamidreza Basseri, Ahmad Raeisi, Mansoor Ranjbar Khakha, Abaas Pakarai, Hassanzehi Abdolghafar

**Affiliations:** ^1^Department of Medical Entomology, School of Public Health, Tehran University of Medical Sciences, P.O. Box 6446-14155, Tehran, Iran; ^2^Malaria Control Section, Department of CDC, Ministry of Health and Medical Education, Tehran, Iran; ^3^Institute of Public Health, Bandar-Abbas Center of Health Research and Education, Tehran University of Medical Sciences, P.O. Box 6446-14155, Tehran, Iran; ^4^Malaria Control Section, Health Center of Sistan-Baluchistan Province, Zahedan University of Medical Sciences, Zahedan, Iran

## Abstract

Seasonal abundance and tendency to feed on humans are important parameters to measure for effective control of malaria vectors. The objective of this study was to describe relation between feeding pattern, abundance, and resting behavior of four malaria vectors in southern Iran. This study was conducted in ten indicator villages (based on malaria incidence and entomological indices) in mountainous/hilly and plain regions situated south and southeastern Iran. Mosquito vectors were collected from indoor as well as outdoor shelters and the blood meals were examined by ELISA test. Over all 7654 female *Anopheles* spp. were captured, the most common species were *Anopheles stephensi, An. culicifacies, An. fluviatilis,* and *An. d'thali*. The overall human blood index was 37.50%, 19.83%, 16.4%, and 30.1% for *An. fluviatilis, An. stephensi, An. culicifacies*, and *An. d'thali*, respectively. In addition, *An. fluviatilis* fed on human blood during the entire year but the feeding behavior of *An. stephensi* and *An. culicifacies* varied according to seasons. Overall, the abundance of the female mosquito positive to human blood was 4.25% per human shelter versus 17.5% per animal shelter. This result indicates that the vectors had tendency to rest in animal shelters after feeding on human. Therefore, vector control measure should be planned based on such as feeding pattern, abundance, and resting behavior of these vectors in the area.

## 1. Introduction

Knowledge of host-feeding pattern and resting behavior of mosquito vectors are important for understanding the host-vector relationship and dynamic of disease transmission and for development of control strategies [1]. It has been observed that vector density and malaria transmission intensity display similar patterns in relation to environmental conditions such as rainfall and spatial and seasonal heterogeneity among shelters [2–4].

However, in spite of more than 45 years malaria-control programming, malaria remains prevalent in southern and southeastern Iran. During the last six years, 15000 to 25000 cases have been reported each year in Iran and more than 85% of them occurred in the south and southeast of Iran (Department of communicable Disease Control). The current annual parasite index (API) is 7 per 1000 inhabitants in the endemic area [5]. Several factors, such as presence of insecticide resistance among vectors [6], parasite drug resistance [7], and socioeconomical problems and population movement [5, 8], have made eradication in this area so difficult. *Anopheles stephensi*, *An. culicifacies, *and *An. fluviatilis* are considered to be primary vectors of malaria in the south and southwest of Iran [9, 10] as well as Indo-Pakistan subcontinent and countries around the Persian Gulf. In some parts of India, these species are responsible for malaria transmission because of their abundance and their potential anthropophily [11, 12]. Anthropophily index (AI) is defined as percentage of blood smears having human blood and is a key factor regarding malaria transmission and may be influenced by abiotic factors, [13], genetics [14], and host behavior. In addition, high densities of mosquito vector enhance vectorial capacity of each species. For example, several epidemics of malaria occurred in southern Iran when the population of *An*. *stephensi* was very high [15]. There is a correlation with density of *An. stephensi*, but not with *An. fluviatilis* for transmission of malaria in Uttaranchal province in India [13], while in Orissa, India, a positive correlation between anthropophilic index of *An. fluviatilis* and the positive malaria cases have been found [12]. The human blood index (HBI) is the proportion of blood smears from a single anopheline species that is positive for human blood [16]. This index is an essential variable in epidemiological investigations of vector-borne diseases such as malaria and also, needed to assess the vectorial capacity of each vector population [17]. A study has been conducted on HBI of malaria vectors based on some environmental factors in Kahnouj district, Southern Iran [18] which showed that *An. fluviatilis *was responsible for maintaining malaria in highland areas. This species also showed high propensity for humans during the warm season in highland Kahnouj district in the south of Iran. However, two studies have been carried out to optimize ELISA and its applicability for identification of anthropophily of malaria vector in Iran [19, 20]. 

Generally endo/exophilic behavior of mosquito is defined based on resting sites of females mosquitoes in indoor or outdoor places after blood feeding. Resting behavior is a very important variable for planning of mosquito control. 

Nevertheless, many developmental modifications have been occurred in the south and southeast of Iran, such as changes in construction of buildings, use of air-conditioning by households, and consequent changes in sleeping behavior of the inhabitant. Mosquito vectors are still able to feed on residents and transmit malaria every year. Therefore, to determine potential of malaria vector biology, dynamics, and to evaluate the risk of malaria transmission, this study was conducted in ten villages situated in mountainous/hilly and plain areas in south and southwest Iran during eleven months and mosquitoes were collected from indoor and outdoor resting places. The human blood index (HBI) among different vectors was determined monthly and compared with seasonality of *Anopheles* spp.

## 2. Material and Methods

### 2.1. Study Areas

**Figure 1:**
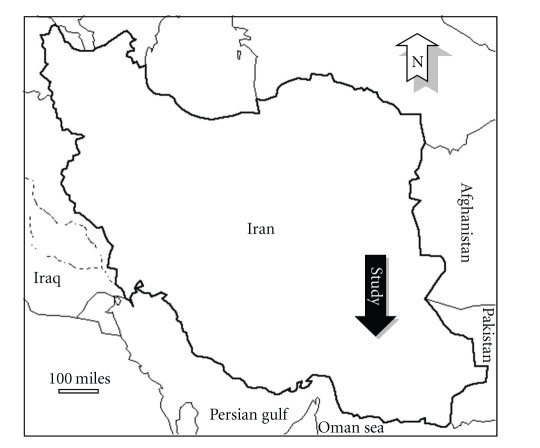
Location of malarious area where the study has been done, in southern Iran.

Geographical characters: the study was carried out in south and southeast of Iran from September 2007 to July 2008 where malaria is still endemic (Figure 1), and based on malaria incidence and entomological indices, ten indicator villages were chosen in two malarious provinces, Hormozgan and Baluchestan. The area is bordered on the north by Zagrus and Mahran mountain ranges, and along the shores of Oman Sea to the south (Figure 1). It is geographically located between latitude 27°50′-26°45′ N and longitude 56°00′-61°60′ E. Generally, the area comprises mountainous, hilly regions in the north, with plains and coastal regions in the south. The coastal and plains regions have a damp subtropical climate but mountainous/hilly regions have a milder climate. Generally, in the coastal/plain regions, the temperature reaches 35–40°C during Summer and near 10°C during Winter. In highland regions, the maximum temperature was 35°C and rarely reached to near 0°C during Winter. In spite of climate variation in the hilly and plain areas, the vectors are present and active in both areas but based on seasonal climate condition, their seasonal activities are varied. Usually, after Winter/Spring begins in south and southern Iran (February, March, and April) and fetches optimum temperature for increasing population of mosquitoes (ranging from 22°C to 30°C), once again after Autumn (September and October) the temperature reaches to optimum condition for mosquito populations to growth. 

 The water sources as breeding places are permanent and temporary rivers, wells Springs, ponds, and pools. The annual rainfall ranged from 80 to 100 mm. The rainfall occasionally received during begging of Summer and Autumn due to monsoon from southeast winds or occurs during Winter due to north winds. Malaria is not equally distributed in south and southeastern of Iran and depending on situation of each area the annual parasite index (API) is varied. Overall in the study areas, annual parasite index (API) was 5.51 per 1000 population in Baluchestan and 3.76 per 1000 population in Hormozgan, respectively, during the study period and the peak period of occurred between May and October, during which nearly 87.4% of the annual malaria cases were reported (Department of CDC, Malaria control section, Ministry of education and Health, personal communication). 

Dwellings were generally constructed of mud and blocks, mostly without screens on windows and doors. The sheds of domestic animals were built close to human habitation and were generally made of mud with straw on the roof or were covered completely with straw and palm leaves. Cow, goat, sheep, and donkey are most common domestic animals in Hormozgan and Baluchestan provinces. The ratios of cow  :  human was ~0.7 : 2.3, sheep or goat  :  human ~5.1 : 1 and donkey  :  human ~0.3 : 1, respectively (Iran Veterinary Organization, personal communication).

The villages were also supplied with electricity and the majority of inhabitants' living rooms were equipped with air-conditioning, which changed their sleeping behavior in Summer. During Winter, the native inhabitants generally slept inside their houses without any protection against mosquito bites, and during mild seasons, they shift outside to sleep but many households did not use bed nets. 

### 2.2. Mosquito Collection

Mosquito collections were standardized as fully described by WHO [21]. During the 11 months, mosquitoes were collected from six indoor and four outdoor shelters in each village as follow.

*Indoor Collections.* The mosquitoes were collected from human dwellings and sheep or cattle sheds by pyrethrum space spray method as fully described by Service [22] and WHO [21]. Briefly, before spray, all the eves, windows, doors, and other exit points in each indoor shelter were closed and then white cloth sheets were spread on the floor. Pyrethrum extract (0.2% in kerosene) was sprayed in the entire space of the room and the room was closed for 15 minutes. After 15 minutes, all the knocked-down insects lying on the cloth sheet were collected carefully with the forceps and placed in petri dishes lined with moist filter paper and brought to the laboratory for further studies. The time of application in each village was early in the morning and during sunrise between 0530 and 0730 h. 

*Outdoor collection* Using Natural or Artificial Shelters. Natural shelters inside and around the indicator villages were periodically searched during early morning and mosquitoes were collected using sucking tube and torch light. In addition, artificial shelters at size of 120 × 120 × 150 centimeters were dug into the ground dimension between feeding place and breeding sites. The mosquitoes were then captured using sucking tube and touch. All mosquitoes were identified based on species keys of Smart [23] and all details of the shelters including kind of habits, temperature, humidity, and date and time of collection were recorded on forms. In addition, collected female mosquitoes were graded to abdominal conditions in each sampling technique as described by WHO [21]. Generally, endo/exophilic behavior of each mosquito species was categorized based on abdominal appearance of the collected mosquitoes as follows. The gravid (G) and/or semigravid (SG) appearance of the female abdomen demonstrate as resting stages of female mosquitoes, and the females with unfed (U) and freshly fed (F) guts are indicative of the seeking stages (seeking for blood meal or resting places). Therefore, ratio of the resting stages classified as tendency to rest in indoor or outdoor places.

Subsequently, blood meal of freshly fed female Anopheles spp. was transferred on a filter paper by squashing the engorged abdomen on the paper. Papers were sealed in plastic pages and kept in −20°C until examined using ELISA.

### 2.3. ELISA Test

Samples were subjected for ELISA as described by Edrissian and Hafizi [19]. Briefly, the dried spots of blood meal were cut out and each put in a well of a micro ELISA plate (NUNC Co, Denmark) and then eluted with distilled water and subsequently a coating buffer (carbonate bicarbonate, pH 9.6) was added. The disc of filter paper was removed and each plate was washed three times with phosphate buffered saline-Tween 20 (pH 7.2). Then 50 *μ*L of antihuman IgG conjugated to alkaline phosphatase were added each well, incubated at 37°C for 2 hr and washed as before. Then 100 *μ*L of substrate solution (1 mg/ml P-nitrophenyl phosphate, Sigma, in 10% diethanolamine buffer pH 9.8 containing 0.5 mmol MgCl2 and 0.02% NaN3) was added to each well and left in a dark chamber at room temperature for 30 min. Finally, the results assessed subjectively by examination with the naked eye or ELISA reader at 405 nm about 30 min after the addition of the substrate solution.

### 2.4. Data Analysis

The data entry was done in Microsoft Excel 2000 and analysis was carried out using statistical package for social science (SPSS) version 10 programme. A *Z*-test was applied to determine differences in anthropophilic index of anophelines in indoor/outdoor places. 

## 3. Results

In total, 7654 female Anopheles spp. including 1330 blood meal fed were captured. The most common species were *An. stephensi* (*n*_total_ = 3411, *n*_indoor_ = 2895, *n*_outdoor_ = 516), *An. culicifacies* (*n*_total_ = 1370, *n*_indoor_ = 1115, *n*_outdoor_ = 255), *An. fluviatilis *(*n*_total_ = 1071, *n*_indoor_ = 888, *n*_outdoor_ = 183), and *An. d'thali *(*n*_total_ = 1764, *n*_indoor_ = 1554, *n*_outdoor_= 210). In addition, 38 females of *An. pulcherrimus *(*n* = 9), *An. superpictus* (*n* = 18), and *An*. *turkhudi* (*n* = 11) were captured, of which only a few were freshly fed and eligible for ELISA examination. Among four dominant vectors, 84.7% (*n* = 6452) were captured from indoor resting places and 15.3% (*n* = 1164) from outdoors. The overall, 1320 females were freshly fed and 302 (22.7%) of them positive for human blood. The overall AIs were 19.8%, 16.4%, 37.5%, and 30.1% for *An. stephensi*, *An. culicifacies*, *An. fluviatilis,* and *An. d'thali,* respectively. The AIs were 5.4%, 2.7%, and 10.4% for *An. stephensi*, *An. culicifacies*, and *An. fluviatilis,* respectively, of the resting populations collected in outdoor shelters whereas it was 14.4%, 13.7%, 27.0%, and 30.1% for *An. stephensi*, *An. culicifacies*, *An. fluviatilis,* and *An. d'thali,* respectively, for resting populations in indoor shelters (Table 1). 

Variation in AIs of *An. stephensi* between indoor and outdoor populations is statistically significant (*Z* = 1.64, *P* < .01). Similar variation was observed between indoor and outdoor populations of *An. fluviatilis* (*Z* = 2.63, *P* < .01) but there was no significant variation in AIs of indoor and outdoor populations of* An. culicifacies* (*Z* = 1.90, *P* > .05). Overall, four species preferred animal sheds more than human houses so that the majority of human blood fed anophelines were captured in animal sheds. Analysis of AIs revealed that population of *An. stephensi* (*Z* = 1.64, *P* < .01), *An. culicifacies* (*Z* = 1.64, *P* < .01), *An. fluviatilis* (*Z* = 1.64, *P* < .01), and *An. d'thali* (*Z* = 1.64, *P* < .01) significantly used animal sheds after feeding on human blood. 

Though very abundant, *An. stephensi* had a relatively low (19.8%) while the less abundant *An. fluviatilis* had a high anthropophilic index (37.5%). *An*. *culicifacies* had the lowest anthropophilic index among four species. The majority of *An*. *d'thali* were captured in indoors rather than outdoors due to high sensitivity of this species to torch light and difficulty to capture with sucking tube (Table 1). This species showed anthropophilic index and also rest in animal shelters.

In addition, female mosquitoes were graded according to abdominal conditions collected indoors and outdoors. The gravid (G) and/or semigravid (SG) appearance of the abdomen demonstrate as resting stages, while the female mosquitoes with unfed guts (U) and/or freshly fed (F) are indicative of the seeking stages. The ratio of resting stages to seeking stages for *An. stephensi* showed that this species had a greater tendency to rest inside (G, SG/E, F = 4.4) rather than outdoors (G, SG/E, F = 2.9). *An. fluviatilis* had a low proportion for endophilic behavior (G, SG/E, F = 2.2) and its preference for resting outside (G, SG/E, F = 7.3) was five times more than *An*. *stephensi* and nearly two times more than *An*. *culicifacies *(Table 2). *An*. *culicifacies* showed slightly more exophilic behavior (G, SG/E, F = 5.5) than endophililc (G, SG/E, F = 3.0). Though the majority of *An. d'thali* was collected in indoor shelters, resting tendency to outdoor (G, SG/E, F = 6.8) was more than indoors (G, SG/E, F = 2.4).

**Figure 2:**
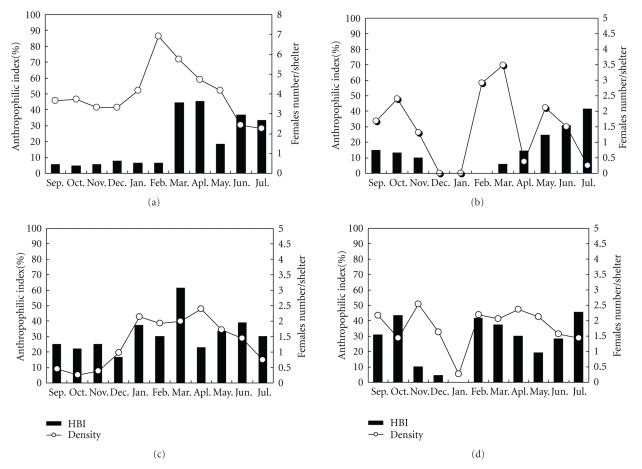
Seasonal anthropophilic index and activities females of four Anopheles vectors in a malaria endemic area in south and southeast of Iran. (a) *Anopheles stephensi.* (b) *Anopheles culicifacies.* (c) * Anopheles fluviatilis*. (d) * Anopheles d'thali. *

The seasonal activities and monthly AIs for the four species are presented in Figure 2. The feeding on human and as well as seasonal activities showed different pattern. *An. stephensi* reached its greatest abundance at the end of Winter, followed by an increasing AI (Figure 2(a)). In contrast, the anthropophilic index for *An*. *culicifacies* was not correlated with abundance (Figure 2(b)). Generally, *An*. *culicifacies,* even at its greatest abundance were less likely to feed on humans than *An*. *stephensi*. The AI for *An. fluviatilis* were higher during the entire year and their numbers indoor places was high during Winter and reached to maximum in March (Figure 2(c)). The AI of *An*. *d'thali* was generally synchronized with its seasonal abundance and was lowest during cold weather (Figure 2(d)). These four species exhibited different seasonal patterns of feeding on humans. 

## 4. Discussion

The present survey revealed that the feeding pattern of four anophelines was varied and also highly depended on seasons changed. In addition, the correlation between mosquito population size and anthropophilic index was different among four vectors. Moreover, in spite of that four species fed on human blood, the mosquitoes prefer animal shelters to rest which this vectors behavior should be highlighted for programs of vector control such as IRS (indoor residual spraying).

 However, environmental impacts as well as host availability affect on anophelines behavior and blood feeding pattern [24]. As results obtained are presented in Table 1, the abundance of mosquitoes fed on human blood was higher in animal shelters than those in human places. This may indicate that these mosquitoes have tendency to rest in animal shelters after feeding on human to complete their gonotrophic cycle. Traditionally, the residents in Hormozgan and Baluchestan areas do not used mosquito net when they sleep in indoor places and this behavior give an opportunity to mosquitoes for feeding on human. However, there is possibility that mosquitoes feed on human in indoor or outdoor places and rest in animal shelters as this behavior was reported in some African species such as *An. arabiensis* [3] or *An. gambiae* [25] where the vectors fed on humans in indoor and rested in animal shelters. 

In the present survey, the combination of mosquito collection from indoor and outdoor places with individual bias towards different vector species helped us to know more about the behavior diversity and densities of the species as well as changing in their blood feeding behavior. We also found that, throughout the study area, *An*. *stephensi* was the most dominant species. This species has been recognized as a chief malaria vector in southern Iran [10, 15] and recently it has been shown that this species has high fitness to transmit *Plasmodium vivax* in Baluchestan area [26]. Previous studies in India also showed that this species plays a major role in malaria transmission in continental India because of its predominantly domestic habits and its behavior [27]. Although *An. stephensi *is primarily a zoophilic species, considerable variability in AI has been reported. For example, in Kolkata, an urban area, AI for this species may reach 100% [28] but in our previous study in Kahnouj District, Kerman province, we observed that the AI was as 0.5% for this species [18] while in the present study the AI reached 19.8%. Kerman province located in north of Zagrus Mountain ranges are with different environment while the south of the mountain ranges (the study areas) is influenced by Oman Sea weather. Therefore, there is significant change in environment as well as inhabitant behavior. This variation of AI may be due to several factors such as environmental impacts and host availability in different provinces with different climate. 

In our study, *An*. *fluviatilis* had a high AI with less abundant. *An. fluviatilis *is now recognized as a species complex comprising at least three sibling species—species S, T, and U [29] but only species T has been reported from Iran [30]. In spite of that species T has been regarded as poor or nonvector in India [31], but it is considered as a main vector in south and southern Iran, Pakistan, and Nepal [30, 32].

It was noted that species T is highly susceptible to malaria sporogony in the laboratory [33]. However, this species is a wild species in Iran and the adult uses any small cavities in the foothill as resting places [6, 34]. The ratio of gravid to semigravid female *An*. *fluviatilis* was comparatively higher in outdoor places (Table 2). It indicates that this species is more exophilic than others. In spite of that this behavior was relatively changed during Winter and population of* An*. *fluviatilis* increased in indoor places (Figure 2). It appears that exophilic behavior of* An*. *fluviatilis* is seasonal dependent and by decreasing temperature, they stay in animal sheds and human dwellings. Similarly, it is reported that environmental impacts influence on resting behavior of *An*. *fluviatilis* as well as feeding behavior in some provinces of India [13, 27, 35]. In addition, by using indoor shelters during cold or mild periods, this species had more contact with humans and therefore it fed throughout the year on humans. Thus, this mosquito can potentially be responsible for maintaining malaria during Winter in south/southern Iran. 

In the present study, the majority of *An*. *culicifacies* was collected in east regions of study area. This mosquito, as well as *An. stephensi* and *An*. *fluviatilis,* is responsible for malaria transmission in southeastern Iran [36]. *An*. *culicifacies* is reported predominantly as a zoophilic mosquito in India [27]. However, we found that this species had relatively low tendency to human blood and mostly fed in July. In addition, based on the ratio of abdominal condition (G,SG/F,E) this species preferred both indoor and outdoor places. Comparable behavior has been also reported from many parts of India [27]. Its greatest abundance of *An*. *culicifacies* was observed in March when the temperature of area was mild, while the maximum AI occurred in July when the average temperatures of local area reached 35–40°C. There may be biotic factors plus environmental impact created anthropogenic environmental modification and impressed diversity in feeding behavior; *An*. *culicifacies* fed most obligatory on human blood. However, we could not find positive correlation between anthropophilic index and population density. 

*An*. *d'thali* has a wide distribution in the foothill areas in southern Iran and is also recognized as a secondary vector for malaria. The first report of sporozoite infection within salivary glands of* An*. *d'thali* was in Bandar-Abbas district, southern Iran in 1972 when malaria was highly epidemic in that area [37]. We found that 30.11% of the population of* An*. *d'thali* was positive to human blood and all captured from indoor places. Adults of this species are very sensitive to light and early disturbed by torch light, this may be the reason we did not collect the adults from outdoor shelters using an aspirator and torch light. Based on abdominal appearance among the collected adults (Table 2), this species had a greater tendency to rest in outdoor shelters. However, *An*. *d'thali* fed on human blood during the year though its AI increased during Spring and Autumn when temperature was mild and the inhabitants did not use air-conditioning in their house without net protection. Thus, the householders' sleeping behavior provides an opportunity for this mosquito to feed on humans too. 

In general, although prevalence of malaria in southern Iran is determined by many factors, our findings indicate that the seasonal activities, anthropophilic behavior, and resting preference of these main vectors favor malaria transmission. In addition, even though some developmental modification, such as supplying electricity to southern Iran, has changed the sleeping behavior of the inhabitants by using air-conditioning and resting inside, they did not significantly influence mosquito and human contact as we observed overall a prevalence of 22.7% human blood among fed females. Even some opportunistic species such as *An*. *culicifacies* which feeds preferentially on cattle, still can be diverted easily to human blood. Thus environmental conditions and lack of knowledge, plus behavior of residents in rural area of southern Iran provide favorable situation for malaria vector to access to human blood. 

At present, vector control activities in the area are mainly restricted to indoor residual spraying (IRS) in selected endemic localities with pyrethroids, larviciding with *Bacillus thuringiensis,* and more recently distributing insecticide-impregnated bed nets by health local authorities [38]. The data of present study show that all species used both outdoor and indoors places for resting (Table 2), therefore, indoor residual spraying may alter behavior of the vectors to rest outdoors. Thus, personal protection can more valuable and reduce AIs in Hormozgan and Baluchestan areas. To achieve this purpose, the communities must be encouraged to use mosquito net correctly. Therefore, local health authorities have to educated resident, and consequently, for evaluating the intervention and change in sleeping behavior, survey on anthropophilic index of vectors is so essential. 

The current national action plan is for total elimination of malaria in Iran [39]. Therefore, to achieve this plan, a precise program which covers all aspects of interactions among vector, humans, and the environment must be additional. In conclusion, south and southeast of Iran have been earmarked for malaria elimination and corroding to data of present study using mosquito nets is more effective and should be highly considered for control of malaria in these areas.

## Figures and Tables

**Table 1:** Analysis of anthropophilic index of anopheline vectors according to type of resting places in south and southeastern Iran (the malaria endemic area) during study 2007-2008.

Species	No. of blood meals				Resting site		
	Out	In	Total	Positive to Human Blood (%)	Out door	Indoors	
						Human dwelling	Animal shed
*An. stephensi*	90	389	479	95 (19.8%)	26 (5.4%)	20 (4.2%)	49 (10.2%)
*An. culicifacies*	57	381	438	72 (16.4%)	12 (2.7%)	13 (3.0%)	47 (10.7%)
*An. fluviatilis*	21	123	144	54 (37.5%)	15 (10.4%)	11 (7.6%)	28 (19.4%)
*An. d'thali*	5	254	259	81 (30.1%)	0	6 (2.2%)	75 (27.9%)

**Table 2:** Abdominal condition of female mosquitoes based on collecting sites in south and southeastern Iran.

Species	Indoor collections		G, SG/F, U (indoors) a	Outdoor collections		G, SG/F, U (outdoors) b	Outdoors/Indoors a/b
	F, U	G, SG		F,U	G, SG		
*An. stephensi*	534 (18.5%)	2361 (81.5%)	4.4	133 (25.8%)	383 (74.2%)	2.9	0.65
*An. culicifacies*	278 (24.0%)	837 (75.0%)	3.0	39 (15.3%)	216 (84.7%)	5.5	1.85
*An. fluviatilis*	279 (31.4%)	609 (68.6%)	2.2	22 (12.0%)	161 (87.0%)	7.3	3.35
*An. d'thali*	455 (29.3%)	1099 (70.7%)	2.4	27 (12.9%)	183 (87.1%)	6.8	2.80

F: Fresh Fed female mosquito.

U: Unfed female mosquito.

G: Gravid female mosquito.

SG: Semigravid female mosquito.

## References

[1] Chaves LF, Harrington LC, Keogh CL, Nguyen AM, Kitron UD (2010). Blood feeding patterns of mosquitoes: random or structured?. *Frontiers in Zoology*.

[2] Kent RJ, Thuma PE, Mharakurwa S, Norris DE (2007). Seasonality, blood feeding behavior, and transmission of *Plasmodium falciparum* by *Anopheles arabiensis* after an extended drought in southern Zambia. *American Journal of Tropical Medicine and Hygiene*.

[3] Mahande A, Mosha F, Mahande J, Kweka E (2007). Feeding and resting behaviour of malaria vector, *Anopheles arabiensis* with reference to zooprophylaxis. *Malaria Journal*.

[4] Mwangangi JM, Muturi EJ, Mbogo CM (2009). Seasonal mosquito larval abundance and composition in Kibwezi, lower eastern Kenya. *Journal of Vector Borne Diseases*.

[5] Raeisi A, Ringwald P, Safa O (2006). Monitoring of the therapeutic efficacy of chloroquine for the treatment of uncomplicated, *Plasmodium falciparum* malaria in Iran. *Annals of Tropical Medicine and Parasitology*.

[6] Djadid ND, Barjesteh H, Raeisi A, Hassanzahi A, Zakeri S (2006). Identification, sequence analysis, and comparative study on GSTe2 insecticide resistance gene in three main world malaria vectors: *Anopheles stephensi*, *Anopheles culicifacies*, and *Anopheles fluviatilis*. *Journal of Medical Entomology*.

[7] Edrissian GH, Afshar A, Sayedzadeh A, Mohsseni GH, Satvat MT (1993). Assessment of the response in vivo and in vitro of *Plasmodium falciparum* to sulphadoxine-pyrimethamine in the malarious areas of Iran. *Journal of Tropical Medicine and Hygiene*.

[8] Basseri HR, Raeisi A, Holakouie K, Shanadeh K Malaria prevention among afghan refugees in a malarious area, southeastern Iran.

[9] Zaim M (1987). Malaria control in Iran—present and future. *Journal of the American Mosquito Control Association*.

[10] Manouchehri AV, Zaim M, Emadi AM (1992). A review of malaria in Iran, 1975–1990. *Journal of the American Mosquito Control Association*.

[11] Kulkarni SM (1990). Density patterns of anophelines and their relation to malaria in Bastar district, Madhya Pradesh. *Indian Journal of Malariology*.

[12] Parida SK, Hazra RK, Marai N, Tripathy HK, Mahapatra N (2006). Host feeding patterns of malaria vectors of Orissa, India. *Journal of the American Mosquito Control Association*.

[13] Devi NP, Jauhari RK (2006). Relationship between *Anopheles fluviatilis* & *A. stephensi* (Diptera: Culicidae) catches & the prevalence of malaria cases at Kalsi area in Dehradun district (Uttaranchal). *Indian Journal of Medical Research*.

[14] Besansky NJ, Hill CA, Costantini C (2004). No accounting for taste: host preference in malaria vectors. *Trends in Parasitology*.

[15] Manouchehri AV, Javadian E, Eshighy N, Motabar M (1976). Ecology of *Anopheles stephensi* Liston in southern Iran. *Tropical and Geographical Medicine*.

[16] WHO [World Health Organization] (1963). Terminology of malaria and malaria eradication.

[17] Bruce-Chwatt LJ (1985). *Essential Malariology*.

[18] Basseri HR, Moosakazemi SH, Yosafi S, Mohebali M, Hajaran H, Jedari M (2005). Anthropophily of malaria vectors in Kahnouj district, south of Kerman, Iran. *Iranian Journal of Public Health*.

[19] Edrissian GH, Hafizi A (1982). Application of enzyme-linked immunosorbent assay (ELISA) to identification of *Anopheles* mosquito bloodmeals. *Transactions of the Royal Society of Tropical Medicine and Hygiene*.

[20] Edrissian GH, Manouchehry AV, Hafizi A (1985). Application of an enzyme-linked immunosorbent assay (ELISA) for determination of the human blood index in anopheline mosquitoes collected in Iran. *Journal of the American Mosquito Control Association*.

[21] WHO [World Health Organization] (1992). *Entomological Field Techniques for Malaria Control, Part I & II Learner and Tutor’s UIDC*.

[22] Service MW (1976). *Mosquito Ecology: Field Sampling Methods*.

[23] Smart J (2003). *A Handbook for the Identification of Insects of Medical Importance*.

[24] Muriu SM, Muturi EJ, Shililu JI (2008). Host choice and multiple blood feeding behaviour of malaria vectors and other anophelines in Mwea rice scheme, Kenya. *Malaria Journal*.

[25] Smith A (1962). Effects of dieldrin on the behaviour of A. gambiae. *Bulletin of the World Health Organization*.

[26] Basseri HR, Doosti S, Akbarzadeh K, Nateghpour M, Whitten MMA, Ladoni H (2008). Competency of *Anopheles stephensi* mysorensis strain for *Plasmodium vivax* and the role of inhibitory carbohydrates to block its sporogonic cycle. *Malaria Journal*.

[27] Dash AP, Adak T, Raghavendra K, Singh OP (2007). The biology and control of malaria vectors in India. *Current Science*.

[28] Hati AK (1997). Urban malaria vector biology. *Indian Journal of Medical Research*.

[29] Subbarao SK, Nanda N, Vasantha K (1994). Cytogenetic evidence for three sibling species in *Anopheles fluviatilis* (Diptera: Culicidae). *Annals of the Entomological Society of America*.

[30] Naddaf SR, Oshaghi MA, Vatandoost H, Assmar M (2003). Molecular characterization of *Anopheles fluviatilis* species complex in the Islamic Republic of Iran. *Eastern Mediterranean Health Journal*.

[31] Nanda N, Joshi H, Subbarao SK (1996). *Anopheles fluviatilis* complex: host feeding patterns of species S, T, and U. *Journal of the American Mosquito Control Association*.

[32] Rao RT (1984). *The Anophelines of India*.

[33] Adak T, Singh OP, Das MK, Wattal S, Nanda N (2005). Comparative susceptibility of three important malaria vectors *Anopheles stephensi*, *Anopheles fluviatilis*, and *Anopheles sundaicus* to *Plasmodium vivax*. *Journal of Parasitology*.

[34] Eshghy N, Motabar M, Javadain E, Manouchehri AV (1976). Biological feature of *An. fluviatilis* and its role in the transmission of malaria in Iran. *Tropical and Geographical Medicine*.

[35] Nanda N, Yadav RS, Subbarao SK, Joshi H, Sharma VP (2000). Studies on *Anopheles fluviatilis* and *Anopheles culicifacies* sibling species in relation to malaria in forested hilly and deforested riverine ecosystems in northern Orissa, India. *Journal of the American Mosquito Control Association*.

[36] Zaim M, Subbarao SK, Manouchehri AV, Cochrane AH (1993). Role of *Anopheles culicifacies* SL and *An. pulcherrimus* in malaria transmission in Ghassreghand (Baluchistan), Iran. *Journal of the American Mosquito Control Association*.

[37] Manoochehri A, Ghiasseddin M, Shahgudian ER (1972). *Anopheles d’thali* Patton, 1905, a new secondary vector in southern Iran. *Annals of Tropical Medicine and Parasitology*.

[38] MOHME (Ministry of Health and Medical Education) Annual report for malaria control program.

[39] Raeisi A Malaria elimination in Iran, progress achievements and challenges.

